# Organizational Changes Needed in Disasters and Public Health Emergencies: A Qualitative Study among Managers at a Major Hospital

**DOI:** 10.1007/s13753-022-00423-4

**Published:** 2022-08-03

**Authors:** Ingela Wennman, Catharina Jacobson, Eric Carlström, Anders Hyltander, Amir Khorram-Manesh

**Affiliations:** 1 Institute of Health and Care Sciences, Sahlgrenska Academy, Gothenburg University, 413 46, Gothenburg, Sweden; 2 Preparedness Unit, Sahlgrenska University Hospital, 413 05 Gothenburg, Sweden; 3 Institute of Clinical Sciences, Department of Surgery, Sahlgrenska Academy, Gothenburg University, 413 45, Gothenburg, Sweden

**Keywords:** Disaster risks, Organizational changes, Public health emergencies, Sweden

## Abstract

Most hospitals have a contingency plan, based on all-risks and all-hazards assessment principles. However, emerging threats and risks often necessitate a flexible approach to emergency management at several levels of a disaster response system, for example, in hospitals. Sweden, and possibly other countries, has limited possibilities of surge capacity in the management of large-scale disasters and emergencies, which necessitates a local/national partnership and a flexible local disaster and contingency plan. This study evaluates the opinions of a selected managerial group, both at operative and strategic levels, regarding possible changes in a major hospital’s contingency plan during the ongoing COVID-19 pandemic. Semistructured interviews were conducted to explore the elements of surge capacity and an operational tool, consisting of command and control, safety, communication, assessment, triage, treatment, and transport. The results show a need to create feasible management methods that can be evaluated, establish clear leadership, put preparedness as a constant point on the highest managerial agenda, improve external monitoring, and create a regional coordinating center. Furthermore, the results emphasize the significant role played by the incident command system and qualified leadership to facilitate competent and crucial medical decision making, as well as to provide reliable communication, collaboration, and coordination in a multi-agency response system during dynamic and unexpected emergencies. These steps enable a constant connection between reactive contingency plans and the proactivity in continuous risk assessment and enhance the flexibility of the contingency plans.

## Introduction

Public health emergencies are defined by their health consequences, as well as their etiology and causes. An event becomes an emergency when its health consequences, scale, timing, and uncertainty have the potential to overwhelm routine capabilities and functions, including healthcare services. This definition is aligned with the hazard approach to preparedness, does not focus only on disasters, and enables capacity development for diverse scenarios (Nelson et al. 2007). Management of public health emergencies, including disasters, hereafter called DPHE (Disaster and Public Health Emergency), focuses on all four phases of the disaster and emergency cycle—mitigation, preparation, response, and recovery (Burkle 2019). As recommended by the World Health Organization (WHO 2019), such management should be proactive. Consequently, measures should be in place to reduce the impacts of an incident, rather than just responding to actual events (Khorram-Manesh 2020a). A proactive approach to DPHE management focuses on the identification of risks and threats to either minimize or eliminate these hazards and encompasses several levels of management and professional areas (WHO 2019). It starts at the family level and expands to the community, regional, national, and international levels, and requires compatibility and harmony in all included measures to support each level by meeting their needs and providing sufficient resources. It also engages diverse professional fields, illustrating the complexity of a DPHE and the need for various skills and diversities (Khorram-Manesh 2020a; Komasawa et al. 2021).

A point of departure of this article was to focus on the complexity of DPHE management and to identify ways of improving it through a step-by-step procedure. A DHPE includes all managerial levels and all professionals who find their roles and responsibilities in an ultimate collaboration and by sharing resources, information, and communication. All participants share the same collective assessment of what has happened and what should be done. Among the different requirements for such a structure, the need for command and control and experienced leadership is evident. A qualified leader is capable of the necessary collaboration and communication to facilitate the correct approach to, and resolution of, medical and nonmedical issues in DPHE, which otherwise may hinder effective management (Phattharapornjaroen et al. 2020; Farcas et al. 2021). The compatibility and harmony needed for a proactive approach necessitate mutual educational initiatives, exercises, and training. These steps should be organized, planned, tested, and evaluated to improve the entire chain of management and facilitate the transfer of knowledge to skills and competence (Khorram-Manesh 2020b).

The term organization refers to “a purposeful hierarchical human system whose members contribute their efforts or other resources to the system to acquire valued resources, such as their livelihood” (Huber and Bartunek 2019). Even if organizations tend to resist changes by building routines, practices, and action patterns that are repetitive (Hinings and Malholtra 2008; Carlström 2012), they are expected to face multiple types of purposes. They may also have a diverse hierarchy, resources, and abilities, and, thus, are exposed to different changes because of internal and external factors, such as the environment in which they interact, and the levels of decision making that guide action. Consequently, the only constant factor for an organization is change, indicating that there is a need for flexibility in an organization to adapt to new changes, risks, and hazards (Morgan 2006; Scott and Davis 2007; Huber 2011; Huber and Bartunek 2019).

Change remains a common experience within healthcare systems globally (Pomare et al. 2019). However, these changes frequently illustrate “physical changes” within the healthcare systems, and behavioral changes are rarely discussed, that is, changes that not only encompass physical changes but also behavioral operational changes, as well as modifications of organizational culture, structural relationships, and roles within national and regional health systems, hospitals, and prehospital teamwork (Fitzgerald and McDermott 2017). Since organizational changes are a necessary part of healthcare during resource-demanding emergencies, there might be a need to change the organizational response system because of new risks and hazards and to adopt new approaches to overcome difficulties that an organization may face in DPHEs.

The occurrence of an increasing number of DPHEs in recent decades might be an indication of either emerging threats or better awareness of the threats but with insufficient management capabilities (Khorram-Manesh and Burkle Jr 2020). The current COVID-19 pandemic might act as a good example, demonstrating both an emerging hazard and the inability of the current contingency systems to provide proper management (Goniewicz et al. 2020). Nevertheless, although plans and systems may have failed to stop the spread of COVID-19, they are still beneficial for other emergencies, since identified changes and acquired awareness of the varying risks and hazards can be used to alter existing plans and organizations (WHO 2019; Choflet et al. 2021).

In Sweden, where the data of this study were collected, there is a limited possibility of surge capacity in the management of large-scale DPHEs. Therefore, there has been a discussion about civilian-military collaboration as part of a new organizational structure, called the “Total Defense Healthcare System” (Khorram-Manesh et al. 2020; Khorram-Manesh et al. 2021). Furthermore, the development and introduction of a new concept, “Flexible Surge Capacity” (FSC), has enhanced the use of all resources within the community to create a new surge capacity by activating all of its elements, that is, Staff, Stuff (devices), Structure (space), and System (Glantz et al. 2020; Khorram-Manesh 2020a). While the concept of FSC has been tested elsewhere (Phattharapornjaroen et al. 2021; Phattharapornjaroen et al. 2022), this study aimed to evaluate the opinions of a selected managerial group, both at operative and strategic levels, regarding possible changes in a major hospital’s contingency plan during the ongoing COVID-19 pandemic. The occurrence of COVID-19 forced the current contingency system to adapt to a new hazard, the pandemic, and simultaneously initiated interagency collaboration, such as civilian-military collaboration, and thus allowed a long-term follow-up of a changing organization.

## Method and Materials

This qualitative study consisted of semistructured interviews with a selected managerial group, both at operative and strategic levels, discussing the elements of surge capacity and an operational tool consisting of command and control, safety, communication, assessment, triage, treatment, and transport. A qualitative thematic analysis was applied to study all accumulated data, focusing on similarities and differences to categorize them into different topics.

### Sample

A group of 15 senior managers, active at both operative and strategic levels, from a hospital (12 males and 3 females), aged 45−69, with previous experience in disaster and emergency management, were included in this study. Similarities in answers increased during the interviews (n = 15) and the 3 last interviews did not add new data to the study. Based on the repetitive answers we considered saturation was achieved and choose to end the data collection (Marshall et al. 2013). All participants were employed by the Western Region of Sweden and had knowledge of and experience in incident management. They were all engaged in the management of DPHEs. All interviewed participants were members of different boards and senior management control teams in the hospital. The hospital chosen for this study is one of the main hospitals in Sweden and one of the largest in northern Europe. Since the turn of the millennium, Sweden has reduced healthcare resources in terms of hospital beds and equipment. Due to increasing political tension in Europe and new threats, however, there is a strong movement to restore the total defense organization in the country, which necessitates a revision of previous contingency plans.

### Process

All participants were invited to a presentation regarding preparedness in general, and particularly in the studied hospital at the beginning of the study in December 2019. This presentation provided the participants with an update on all the most recent and correct information about their hospital’s preparedness and various risks and hazards that they may face in the future (a mutual situation assessment before the study started). All participants had access to a recorded version of the presentation and were informed that the first author would contact them for in-depth interviews in 2020. The purpose of the interviews was to collect the necessary information for a new, revised, contingency plan. The study continued under the COVID-19 pandemic and ended in September 2021.

### Ethics

All participants freely volunteered to take part in this study and could withdraw at any time without penalty. They received information relating to the study’s purpose, the voluntary nature of participation, absolute confidentiality, anonymity, and secure data storage. Verbal and written consent was obtained from all participants. The study complied with the ethical guidelines and principles stipulated by Swedish law. In Sweden, ethical approval is mandatory if the research includes sensitive data regarding the participants, such as race, ethnical heritage, political views, religion, sexual habits, and health or physical interventions, or employs a method that aims to affect the person physically or psychologically (Swedish National Constitution 2003).

### Interviews

All participants took part in the interviews, digitally or physically, lasting around 45 minutes. Interviews started with the renewed presentation of the facts about the hospital’s preparedness and possible threats, followed by two open-ended questions, which should be answered by considering three levels (micro, meso, and macro) to obtain a comprehensive perspective on the status of the response system: (1) Existing shortcomings in the current contingency plan before, during, and after an incident? (2) Solutions/suggestions for mitigating/eliminating existing shortcomings to improve the contingency plan?

Academic experts in disaster medicine and crisis management scrutinized the questions intended for use in the interviews to improve their validity. The interviews were updated in terms of clarity, accuracy, and comprehensibility. To test the feasibility, logic, and perception of the questions, two of the authors conducted a pilot interview. The interviewees in this pilot study were not included in the final version of the manuscript but were senior management members equally competent and experienced in the subject as those included in the study. All authors approved the final list of questions after some modification, based on the results of the pilot study.

The first author interviewed all participants. During the interviews, interviewees were asked to present their views about the current contingency plan, discuss the need for potential changes, and suggest new changes, if deemed necessary. The interview environment was intended to be friendly to allow for critical comments and suggestions. All interviews were recorded, coded, and transcribed for further analysis.

### Data Collection and Analysis

The first two authors, initially, analyzed the collected material after reading the accumulated data several times. All authors read the results and gave their feedback during several meetings. This process aimed to validate the collected data, resulting in the analyses. The collected data were divided into two groups, based on the responses to the questions. All statements and opinions were gathered and analyzed separately in each group.

A qualitative thematic analysis was applied. This content analysis addressed all accumulated data, focusing on similarities and differences to categorize them into different topics. A consensus was reached to create new themes (Erlingsson and Brysiewicz 2017), categorized according to the vital elements of surge capacity, that is, Staff, Stuff (devices), Structure (Space), and System (Khorram-Manesh 2020a; Brenna and Das 2021; Phattharapornjaroen et al. 2021).

## Results

The results of the interviews are presented below, divided into two major sections (Sects. 3.1 and 3.2). Initially, the interviewees described the existing shortcomings in the current system before, during, and after a disaster or an emergency. Furthermore, the participants offered their solutions to the existing insufficiencies in the second section of the results.

### Existing Shortcomings in the Current Contingency Plan

Initially, each participant was asked to present and discuss existing shortcomings in the current system during the dynamic phases of disaster management as what was missing before, during, and after an incident.

#### Before an Incident

There are several important factors before an incident that influence the outcome of the incident management. These are a part of the preparedness phase and should be carefully planned and tested.

*(1) The unclear role of a preparedness organization in the current hospital management system.* Most of the respondents declared that incidents that could trigger a contingency plan are rare. Consequently, questions referring to a plan’s quality and efficiency end up in the daily issues at a healthcare facility and create difficulties as to what should be prioritized, namely current problems or those related to what may come in the future. In addition, according to the respondents, it was extremely difficult to become competent in what happens very seldom. The following reported shortcomings were, however, important to address and define in an improved version of a contingency plan: the relationship between the current line of hospital managing organization and the preparedness organization, their roles, and responsibilities, and the mandate they may need in order to execute their specific actions and measures. It was considered by surveyed managers very important that the management organization had on its team a representative from the in-house preparedness organization.

*(2) Endurance.* According to the respondents, the hospital may only be able to receive a large number of disaster victims for a short period due to the lack of institutional endurance and resiliency. However, the majority recognized the lack of reserve material due to the present concept of Just-In-Time delivery, when all storage units have enough materials for one week.

Often, there is no real-time control of what may be missing. Solving these issues requires a decision on what ability should a hospital have; a larger incident will overwhelm our capabilities, even regionally. (Senior manager, university hospital)

Almost all the participants acknowledged that there is a lack of continuity, which is needed to secure the functionality of the healthcare system in a large-scale incident. It was not clear whether an IT (Internet Technology) incident could be managed by manual routines and procedures. The communication needed between all levels of management and the delivery of needed material had to be secured. The participants emphasized that the functionality of the organization in an environment with no access to technical devices, particularly in emergency cases, should be guaranteed. The threats confronting the hospital’s functions should be analyzed, and the risk and vulnerability analysis had to be conducted routinely.

We need to collaborate with other organizations and our military healthcare system. The current discussion regarding civilian-military collaboration should lead to a consensus on how we may be able to collaborate in crises, armed conflicts, and disasters. (Member of a hospital board)

*(3) Collaboration with other specialties—Insufficient perception of the civilian-military collaboration in emergencies.* Several participants noted that decisions have been made for a civilian-military collaboration within future total defense healthcare. But this is difficult to achieve because there was no synchronization or compatibility between the two healthcare organizations.

There are special circumstances regarding the preparedness level in an armed conflict; however, the civilian organization lacks the insight and knowledge about the requirements for command and control in such an incident. There is a difference between collaboration needed in an armed conflict compared to that needed during peace when all collaborations are made according to the existing rules, laws, and agreements. We must be aware that in such a situation, existing resources and those available after a second surge capacity would be very limited. (Senior hospital physician)

Financially, it was regarded as a dilemma to determine how much investments should be set aside for preparedness and civilian-military healthcare collaboration.

How much should we invest in something that may not happen but needs to be managed adequately when it happens? Better collaboration with other specialties, particularly primary and community healthcare, must be established. The involvement of voluntary organizations is necessary. (Senior hospital manager)

*(4) A central function with the capability of having a total overview of the processes is necessary.* The majority of the participants reported a need for a central unit, particularly at the regional level, with the responsibility to overview an incident, and the ability to estimate the needs and the availability of resources through communication with all facilities within the region.

Such a function prevents all misunderstandings and parallel tasks that may consume all resources and have an impact on society and victims. (Senior hospital physician)

*(5) Exercises and training. *It was considered crucial to allow time for exercise and training as an important part of preparedness. The entire organization needed to understand how to handle a dynamic incident during a longer period and realize its consequences for the staff and the functionality of the facility. The majority of the participants believed that training and exercises are necessary for organizations to get to know each other and to enhance collaboration.

Working in diverse positions with different responsibilities, training, and exercises with other organizations make future contact easier during an emergency. Additionally, exercise and training facilitate better communication and help to achieve a better understanding of limitations and capabilities, which enable correct decision making in a consensus. Otherwise, there may be a disagreement and discrepancy in the assessment of a situation between the managerial leadership and the contingency leadership on what should be done. Better communication at the operational level, facing all issues, is necessary. (Senior hospital manager)

#### During an Incident

Almost all participants emphasized the significance of a continuous process in the management of disasters and emergencies. It was clear that a good start before an incident is a necessity; however, to maintain the process several steps are needed.

*(1) The roles and responsibilities.* Although the people involved in an organization may have defined roles and responsibilities before an incident, the participants admitted that they have been involved in situations during an incident that not only exceeded their qualifications but also necessitated assuming diverse roles and responsibilities in a dynamic process. One person also shared about having to play several roles and responsibilities because of the lack of staff. The participants declared a need for clarification of the inter- and intra-organizational roles and responsibilities. The following statement clarified the significance of defining one’s role and responsibility as a crucial element in what mandate individuals have and how they can collaborate with others.

Within the medical facility and the regional network, there are those responsible for resources and other measures during all phases of an emergency, who do not know their responsibility, mandate, and organizations with which they may need to collaborate. Another significant issue is the way some people may possess a role and take the responsibility for a managerial level that they are not qualified for during a major incident. (Senior hospital physician)

*(2) The design of a contingency plan, method, and tools.* The participants underlined the need to work and make decisions based on rational thinking compared to routine intuitive and emotional management in peacetime. They emphasized the need for a contingency plan that covered risks and hazards and encompassed certain functions necessary for managing an incident. The following statement affirms that a contingency plan should not be designed based on how it may fit the existing organization, but rather should be based on what it requires to be successfully implemented and how it sufficiently confronts all risks and hazards.

The focus must be on the incident and its outcomes. Particularly important is the way an incident command system works. It should not be a question about methodology but a focus on the incident. (Senior hospital physician)

Furthermore, the participants asserted that significant issues might appear during the management of an incident when the tools and methods utilized were not adjusted to the actual situation and not recognized by the involved organizations. The distribution of resources and medical triage might be two good examples. According to the participants, crucial decisions regarding triage and resource distribution required a clear and transparent process and a known decision maker.

*(3) The level of preparedness.* The participants regarded choosing the correct level of preparedness and thus using enough staff and stuff as the two most important factors to successfully manage an incident. For several participants, it was unclear when and at which level the contingency plan should be activated.

It is important that decisions made on the level of preparedness and the functional activity of the facility are not questioned and criticized afterward, since these decisions are made based on the presented facts and can hardly be reconstructed after the incident. (Senior hospital manager)

Several participants underlined their conviction that there is often one single opportunity to save the situation and the victims. Overestimation may happen but should be recognized as the best choice in a situation when several people may die due to resource scarcity and inability to react.

*(4) The line of hierarchy.* According to the participants, in a flat and horizontal organization, there might be an issue with implementing the decisions made by a higher level of management. Therefore, all leaders in the routine managerial hierarchy should learn to respect and implement decisions made by the command incident committee.

In hospitals with several complexes, there might be several committees. It is important that the line of hierarchy is clear to facilitate a quicker and more adequate decision. A simpler organization might have better functionality. Several leadership levels may also consume personal resources and, particularly, experienced leadership that can make vital decisions. High-level clinicians may have a better place in the care of patients. (Senior hospital manager)

Simultaneously, the majority of participants could recognize the need to create a better working environment, where the staff involved could help each other, irrespective of their positions, background, and level of knowledge. Thus, better coordination was deemed necessary. Although an incident command system requires some levels of hierarchy, several participants expressed the view that coordination and cooperation should be enhanced to utilize and distribute all necessary resources and receive a better understanding of resource requirements and decisions made.

*(5) Information and Communication. *Almost all participants emphasized that collaboration, cooperation, and coordination require good communication when all involved staff, facilities, and organizations, especially service and maintenance units, could speak the same language and communicate accordingly. Several participants mentioned:

It is very easy to forget the victims’ relatives and the psychological impact an incident may have on the victims, their relatives, and staff. (Member of a hospital board)

As mentioned by many, information sharing was regarded as an important factor in incident management. Authorities and facilities should communicate and share information with the affected population.

#### After an Incident

There are several steps that should be considered in the post-incident period to guarantee the development of the guidelines and instruction, using the outcomes and lessons learned.

*(1) Take home messages and lessons learned.* The participants underlined those new strategies that should be employed to learn from the mistakes made.

Lessons learned should be shared with a transparency and without any hesitance for punishment or other employment-related consequences. (Member of a hospital board)

One participant underscored that learning from real and actual events might be the only chance a hospital can have to improve its response to subsequent incidents.

*(2) Evaluation and improvement.* Most participants agreed that the ability to receive and accept comments, critiques, and evaluation results following an incident is a necessary step to improve the contingency plan and mitigate or eliminate all shortcomings.

### Solutions/Suggestions for Mitigating/Eliminating Existing Shortcomings to Improve the Contingency Plans

Each participant was offered time for reflection to suggest how the aforementioned shortcomings and insufficiencies could be mitigated or eliminated. Table 1 illustrates all 225 citations/suggestions compiled into 12 diverse categories, based on detailed content analysis. The aggregation of all suggestions from Table 1 was further compiled into six topics (1−6) below.

**Table 1:** Suggestions and solutions to the shortcomings identified in a major hospital contingency plan evaluated in this study from 15 respondents (225 citations proposing solutions)

42 citations in total: Communication ways, Lessons learned, support, and collaboration with other agencies (several mutual citations)	Collaborate with nonprofit and voluntary organizations. Learn from other agencies, such as the military and private healthcare organizations. Establish connections with other agencies in peacetime to enhance the partnership in the event of a crisis. Be agile in collaboration. Ensure that information flows into the hospital through others. Recognize and confirm in peacetime how and what other organizations can contribute and what responsibility they have in crises. Coordination in standby mode. Demand more control and leadership from the regional command center. Request regional centers’ continuous collaboration with the national crisis organizations. Alignment is needed. Request collaboration with the regional IT center and other competencies. Demand national coordination to prevent different approaches by diverse organizations. Collaborate with primary healthcare centers. Collaborate with centers having Internet and cyber knowledge. Clarify the means of having contact in peacetime.
29 citations in total: Governance, leadership, and mandate	In the event of an incident, the routine leadership must lead the operational work, and employees with a mandate must be able to propose solutions. Necessary agreements for service provision with supporting agencies should be signed and sealed in peacetime (for example, medical devices and pharmaceutical agents). Clarify responsibilities and roles; give a mandate to the managerial line at an event. Do not trigger the staff mode when not needed. We need proximity to businesses. Bus companies must operate within minutes, be strategic, and offer transportation services continuously. Strong manager/line structure in the event. Senior executives should have the highest responsibility. The working situation should be as similar to the normal mode as possible. The principles of responsibility, proximity, and equality must prevail in all our planning. Strengthen communication in the organization between the micro-, meso-, and macro-levels.
10 citations in total: Guidelines and routines	Revise existing documents, such as the disaster log. Create templates to support the committee’s work. Resolve unnecessary hierarchies and power imbalances between professions, gender, and so on. Produce and live by the approved documents regarding crisis preparedness. Ensure needed rules and routines. Make fewer, more compatible governing documents. National guidelines regarding forms of care are required in the event of war.
4 citations in total: “Think out of the box”	Take advantage of people who think differently. Diversity may offer new solutions. Support creativity and change of priorities. Sometimes we may need to live outside our comfort zones to solve problems in a crisis. Creativity and proactivity are in demand.
44 citations in total: Education, exercises, and training	Continuous exercises for the disaster management committee. Skills development for all staff. Collaborative exercises with other agencies, such as the Armed Forces. Ensure that leadership has good management knowledge. Management needs knowledge about crisis preparedness and their roles. Time must be allowed for practice. Simulation exercises in unexpected events. Face-to-face exercises, interactive exercises. Understand what the consequences are when IT is down. Individually tailored education. Identify shortcomings by doing exercises. Train alarm chains. Train in civil defense. Short training versions. Practice communication so that everyone speaks the same language. Ensure (make it mandatory) that everyone has received training. Develop a training plan.
8 citations in total: Information to staff and citizens	Have a communication plan (partners and citizens). Evaluate the amount of information to be released. Transparent communication. Secure hospital reputation by creating trust through correct information. Inform citizens what to do and where to get information. Information channels (short messages!).
12 citations in total: Digital support	IT support during the operative management phase. One working platform for all involved organizations. App-solutions to enhance educational and training initiatives and exchange of experience and knowledge. Create an information system to assess resources, maintenance, number of, and follow-up of patients during an incident. Mass Casualty Communication. Communication transformation between diverse management levels. Bed occupancy in real-time.
16 citations in total: Robust organization	Evaluate hospital command group. Ensure sufficient human resources in the command group over time. Need to focus on and be prepared for events that never happen. Ensure the hospital’s functionality, for example, in the event of malfunctions. (Can be the number of staff, infrastructure, and so on.) Ensure manual routines exist to avoid system collapse. Have plans for events that are protracted. Knowledge must not be personal but must be linked to function. Strategic services/people must handle several different roles. The ability must be 24-7. Have a continuous routine for monitoring the outside world.
24 citations in total: Verify/qualify which functions are needed in crisis management	Appropriate background and competence. Personal appearance. Identify staff with special knowledge. Need competences in partners who can support the process, such as service units. Lawyers and those with knowledge in medical technology? Human Resource Specialist to recruit suitable workforce. Runners as a communicative way. Preparedness Officer in Charge with knowledge of potential complexity and diverse networks.
6 citations in total: Evidence-based working	Review working conditions in the command group to identify necessary methods for work and research. Compare the current system with other countries. Design management methods suitable for how people react. Research and evidence-based methods. Implement nationally recommended working methods.
22 citations in total: Evaluate, give feedback, learn from risks and hazards, and implement necessary measures	External evaluation. Make decisions based on risk. Use experiences during training and change the organization. Change what is necessary to change based on the evaluations. Use necessary and validated tools to improve an organization. Learn from risk analysis. Share the results of evaluations with everyone. Make plans real.
8 citations in total: Secure mutual assessment	Create a mutual arena for discussion and exchange of ideas. Consider and discuss cultural issues. Partners need to have a mutual assessment and opinion on how resources are to be used. Command group meetings should end with a consensus over what were the outcomes and which decisions have been made. Avoid acronyms. Everyone should know the methods of working and work accordingly.

### Feasible and evaluable methods

Almost all participants recognized that the lack of feasible and evaluable measures and methods was a significant shortcoming. To manage this point, they proposed that organizations should use methods and measures that are easy to implement and their outcomes are easy to evaluate, by following the following steps:

- Adopt standardized measures for prevention/elimination of risks and evaluation methods and instruments to control outcomes. The evaluation should be systematically conducted by independent organizations. Recommendations following an evaluation need to be implemented accordingly.
- Create educational initiatives that establish bridges between organizations, locally, regionally, nationally, and even internationally. An educational system that can prepare all staff, including new employees, continuously on what a major incident is, how it is managed, and how the managing organization can be formed is essential.
- Learn in a collaborative mode from other agencies, such as various voluntary organizations and the military. The armed forces, for instance, have a better experience with such a methodology. They utilize scenario-based strategic plans. They analyze an incident and develop their new strategy around what has happened, including shortcomings and successes.
- Promote a transparent organization that shares its experience from lessons learned with other agencies. One way might be to have representatives from different agencies in the incident command group. Several occasions for discussion and information sharing with other organizations are necessary.

#### Clear leadership

According to the participants, there was a need to create and define a clear leadership in an incident to control and have an overview of the implementation process and the steps mentioned in point (1) above, by clarifying and defining:

- Who is responsible for decision making (not every step can be planned in advance).
- How educational initiatives and opportunities can be offered to equip all involved staff to be aware of cultural, social, medical, and nonmedical issues that they may encounter during crises.
- How a mutual goal can be achieved by collaboration and through the contingency system.
- How to share information with other agencies and the affected population.

### Make preparedness issues part of the agenda for the highest managerial level

All participants underlined that the lack of continuity in discussions regarding the importance of preparedness at the highest level of hospital management was another vital issue to address. If preparedness and the necessity of being proactive in response were not discussed continuously, according to the participants, it would result in inappropriate preparedness. Therefore, they suggested the following steps:

- Raise questions and issues regarding preparedness on the agenda at the highest managerial levels continuously.
- Make the highest managerial level aware of the important elements of disaster management and surge capacity and the ways they can collaborate locally, regionally, and nationally if needed.
- Bring up the necessity for exercises and training.
- Create an organization with the right staff on the right spot and with the necessary mandate to make vital but necessary decisions.

#### External monitoring

One significant part of preparedness, as stated by the participants, was being aware of what happens regionally, nationally, and globally. Therefore, several participants highlighted the importance of performing continuous external monitoring by:

- Following up on what happens locally and regionally.
- Learning and making it mandatory to perform risk and vulnerability analyses.

#### Creating an organization with better planning for resource management and logistics

The majority of the participants regarded all four suggestions in section (3) as necessary elements to develop an organization that is fully aware of its capabilities and limitations, as well as the risks and hazards that might overwhelm its structure and could allow an organization to:

- Plan for resources that might be needed in an emergency, based on risk and vulnerability analyses.
- Consider logistics and maintenance issues carefully by committing the hospital and external distributors or companies to a signed agreement for delivery under specified situations.
- Guarantee patient safety by appropriate and necessary documentation of disaster victims within the current medical file system or as an external disaster journal, digitally and/or in paper form.
- Prepare an organization that can work proactively, but can also make decisions reactively.

#### Creating a regional coordinating center

Several participants emphasized that the entire process of incident management involves diverse organizations and perhaps different locations. This may indicate a need for a central management level that could have an overview of what is going on and be capable of distributing resources in a way where a balance could be achieved between the needs and ability, without often overwhelming, the nearest hospital. A plausible solution that was suggested was a regional center since it could:

- Have an overview of resources and the responsibility for coordination of actions and resource distribution.
- Offer a possibility for collaboration with others by fully following up on the balance between needs and resources.
- Unburden medical facilities by doing external monitoring, national and international communication, and large-scale collaboration with other agencies.

## Discussion

The novel outcomes of this study are twofold: (1) The study offers a long-term follow-up of changes in an organization in real-time instead of dependence on lessons learned; and (2) it recognizes the need for both reactive and proactive elements in a contingency plan to face emerging risks and threats.

This study started at the end of 2019 and aimed to evaluate the opinions of a selected managerial group, with experience both at operative and strategic levels, regarding possible changes in a major hospital’s contingency plan during an emerging disaster/pandemic, and the use of civilian-military collaboration in managing such an event. The total defense system in Sweden is defined as a system in which both the civilian and the military healthcare systems are included, but the responsibility of providing healthcare lies on the civilian side (Khorram-Manesh et al. 2020). Such an adjustment, during a time when economic constraints have already influenced the civilian healthcare system, is not an easy task (Khorram-Manesh 2020a). While interviews were initiated, the COVID-19 pandemic created a realistic picture of how an affected contingency plan may need to adapt to new and emerging threats. Consequently, the perceptions of what to change to be prepared became a reality. Therefore, in contrast to other studies, this study offers a long-term follow-up of an organization in crisis and identifies needed changes prospectively, that is, learning by doing, such as regular training exercises, instead of lessons learned by retrospective analysis (Yi et al. 2010; Adini et al. 2014; Choflet et al. 2021).

Looking at the outcomes of this study, the comments provided a data matrix for achieving reliable preparedness before, during, and after a major incident that could be merged into a few crucial topics. These topics are: command and control, endurance, collaboration, communication (information), the need for a central coordinating function, education, exercises and training, and a standardized methodology for response and evaluation. There is nothing novel about these categories, and they have been listed in several previous publications (Lowes and Cosgrove 2016; Khorram-Manesh et al. 2017; Phattharapornjaroen et al. 2020). Their presence in this article, expressed by people who play a major role in crisis management and who have been working with its diverse strategic and operative aspects during the two years dominated by the COVID-19 pandemic, indicates the lack of preparedness, despite Sweden having a functional preparedness system. This may imply that proactivity, as required by WHO, is not enough and there is also a need for reactivity in the dynamic process of DPHEs. Another compelling point in this study is the fact that the topics are all associated with three elements of surge capacity (Khorram-Manesh 2020b; Khorram-Manesh and Burkle Jr 2020), that is, staff, stuff, and system but NOT with structure and space, which may normally be perceived as a major obstacle to handling an emergency in a hospital (Yi et al. 2010). It is then logical to perceive that an emergency needs personnel, devices, and guidelines, but not necessarily spaces.

Most of the comments, in all phases of management, concern the organizational hierarchy and functionality of incident command systems. Comments on the need for clarification of the leadership, the roles, and responsibility, the chain of commands for decision making, the inclusion of the preparedness organization into the ordinary managerial system, including representatives from various agencies for better collaboration, and so on, are all in need of functional and trained leadership in an organization. This crisis management institution must be willing to change and improve, understand the need for reasonable safety for patients and victims, and provide the necessary proactive and reactive steps to execute vital decisions (Al-Sawai 2013; van Rossum et al. 2016; Phattharapornjaroen et al. 2020). These objectives include realizing the importance of collaboration with other agencies, coordination of resources, cooperation within and outside the hospital, the need for continuous maintenance, communication and information sharing, and all medical and nonmedical issues that may require an uncomfortable but necessary medical decision-making process (Khorram-Manesh et al. 2017; Brenna and Das 2021). The leadership, aware of the consequences of a major incident, must equally prepare its organization, physically and mentally, for the significance of such decision making and its impact on triage, treatment, and transport opportunity, as well as other decisions that must be followed and executed for the best of all and not just a few. Adequate knowledge of cultural and social issues, which may influence the execution of tasks, needs proper consideration to create an environment and opportunity to move everyone toward a mutual goal. Sharing information with all managerial levels within the medical facility, as well as with collaborative agencies and the affected population outside the hospital, is another critical characteristic of effective leadership (Maiers et al. 2005). In many cases, information should be delivered in collaboration with other agencies, such as the rescue teams and police.

A qualified leadership incorporates preparedness issues into the managerial agenda continuously. This may include placing a representative from the preparedness organization in the higher managerial leadership, not only to highlight the significance of the preparedness task, but also to increase the validity and value of decisions made due to continuous external monitoring of local, national, and global events (Adini et al. 2014). The highest managerial level needs to know all important elements of disaster management, surge capacity, and how they need to collaborate, locally, regionally, and nationally. Finally, higher managerial levels need to recognize the need for education, training, and exercises for key persons in the management system to gain the necessary knowledge to prepare colleagues to manage unexpected incidents and to develop the required instruments for communication, coordination, cooperation, and collaboration (Khorram-Manesh et al. 2016a; Goniewicz et al. 2021; Khorram-Manesh et al. 2022). Thus, developing an organization with the right staff on the right spot and with the necessary mandate to make vital but necessary decisions is imperative.

To achieve these points, there is a need for providing educational initiatives that build bridges between various organizations, locally, regionally, nationally, and internationally (Ingrassia et al. 2014; Khorram-Manesh et al. 2016b). Continuous exercises and training are necessary for an environment in which planning aims at proactivity in the management of DPHEs, and mistakes are allowed to develop reactive decision-making ability, with no harm to victims, within the organizations and among various agencies. Training exercises also help to identify shortcomings in logistics and maintenance issues; the lack of external delivery of medicine, food, and instruments; documentation; and other insufficiencies where their identification would contribute to more effective planning (Khorram-Manesh et al. 2016a).

Having representatives from several important agencies in a collaboration exercise provides opportunities and occasions for agencies to share their experiences and inform each other about the lessons they have learned during their management of an incident. Such discussions result in the organizations revealing their limitations and capabilities concerning staff, skills, and resources, and what each organization can expect from the others (Berlin and Carlström 2015). It may also lead to the creation of plans and contact avenues through which diverse organizations can effectively initiate their plan alone and in collaboration with others. As noted by some participants, the armed forces possess better experience in the “lessons learned” methodology, and they analyze the outcomes of an incident to adjust their strategic plan for diverse scenarios (Rhomberg 2000). Since civilian-military collaboration is desired and one of the most suitable partnerships between two healthcare organizations in Sweden and elsewhere, the armed forces could perhaps share their knowledge and experience or offer opportunities for joint analysis of civilian incidents. Such collaboration could also ease the formation of the future Total Defense Healthcare System (Khorram-Manesh et al. 2020; Khorram-Manesh 2020b; Khorram-Manesh et al. 2022).

Exercises and training occasions could equally serve as evaluation methods for further improvement of contingency systems. They could simply provide an opportunity for risk and vulnerability analysis or be regarded as occasions when lessons learned can be discussed transparently to refine and revise a contingency system (Khorram-Manesh et al. 2016a). Finally, they could also display and emphasize the significance of having a central coordination center, especially in complex scenarios, when an all-embracing command group is needed to assess the received information; initiate cooperation, coordination, collaboration; and maintain a solid communication between several medical facilities and other agencies (Khorram-Manesh et al. 2009). Additionally, exercises and simulation training can be used to evaluate the feasibility of using the results of this study (possibilities and difficulties) and the changes in contingency plans in other areas.

## Limitations

This study has some limitations. To begin with, the study period has been long, with both positive and negative impacts. Although this long period offered a good opportunity for a prospective observation of changes, the daily impact of the pandemic may have influenced the participants’ opinions.

One of the researchers conducted all the interviews. Although this makes the standardization of the interviews easier, it may also be biased by having only an individual’s interpretation. However, two researchers have analyzed the collected data, and the entire group of researchers has evaluated all the results.

Another limitation can be the differences between physical and digital interviews. Digital interviews, besides being cost-effective, enhance long-distance participation. However, the quality of interviews may be affected by technical difficulties. Another significant difference is the fact that body language is easier to capture in physical, in-person, interviews, while it can be difficult to read visual cues in a digital interview. We believe that the negative impacts of digital interviews are negligible in this study’s selected group of participants (Basch and Melchers 2021).

Finally, this research is done in Sweden and under reigning Swedish conditions, and comparative studies are missing. Thus, it might have some limitations to apply in other countries. However, the outcomes and take-home messages of combining the current reactive plans with proactivity in identifying emerging risks to create more flexibility in contingency plans are transferable to other similar settings.

## Conclusion

This study aimed to evaluate the opinions of a selected managerial group engaged in both operational and strategic responsibilities that involved contingency preparation for possible changes in a major hospital’s contingency plan during the ongoing COVID-19 pandemic. The results emphasize the significant role of proactivity in planning for an incident command system, qualified leadership, and a reliable management system. However, the findings also show that such leadership needs to facilitate qualified medical decision making, communication, collaboration, and coordination through a reactive approach. Both elements of proactivity and reactivity are needed for the establishment of a reliable link between agencies, employees, and the public to guarantee the execution of decisions made and tasks assigned. These steps in DPHE management can be trained and practiced, using simulations and other types of educational initiatives to increase the awareness of all involved agencies and the affected population.

Since most contingency plans are “reactive plans,” the major implication of this study is to recognize the need for proactivity in DPHE management. One suggestion is to bring up and discuss the preparedness issue as one of the main points on the agendas of all healthcare facilities. The goal of this practice is not only to discuss the emerging risks but also to enhance the flexibility of all contingency plans.

The only constant factor in life is change. An increasing number of disasters and public health emergencies necessitates better planning for future incidents by structuring current contingency plans to both foresee the upcoming threats and enable multiagency collaboration. The current study examined the needed changes for multiagency collaboration, but it could also follow all the needed changes to manage the current COVID-19 pandemic prospectively.
